# Phycotoxins in Marine Shellfish: Origin, Occurrence and Effects on Humans

**DOI:** 10.3390/md16060188

**Published:** 2018-05-29

**Authors:** Federica Farabegoli, Lucía Blanco, Laura P. Rodríguez, Juan Manuel Vieites, Ana García Cabado

**Affiliations:** Food Safety and Industrial Hygiene Division, ANFACO-CECOPESCA. 16, Crta. Colexio Universitario, 36310 Vigo (Pontevedra), Spain; ffarabegoli@anfaco.es (F.F.); lucia@anfaco.es (L.B.); lauraperez1982@hotmail.com (L.P.R.); direccion@anfaco.es (J.M.V.)

**Keywords:** shellfish toxins, biotoxins, HABs, DSP, PSP, ASP, NSP, toxic phytoplankton

## Abstract

Massive phytoplankton proliferation, and the consequent release of toxic metabolites, can be responsible for seafood poisoning outbreaks: filter-feeding mollusks, such as shellfish, mussels, oysters or clams, can accumulate these toxins throughout the food chain and present a threat for consumers’ health. Particular environmental and climatic conditions favor this natural phenomenon, called harmful algal blooms (HABs); the phytoplankton species mostly involved in these toxic events are dinoflagellates or diatoms belonging to the genera *Alexandrium*, *Gymnodinium*, *Dinophysis*, and *Pseudo-nitzschia*. Substantial economic losses ensue after HABs occurrence: the sectors mainly affected include commercial fisheries, tourism, recreational activities, and public health monitoring and management. A wide range of symptoms, from digestive to nervous, are associated to human intoxication by biotoxins, characterizing different and specific syndromes, called paralytic shellfish poisoning, amnesic shellfish poisoning, diarrhetic shellfish poisoning, and neurotoxic shellfish poisoning. This review provides a complete and updated survey of phycotoxins usually found in marine invertebrate organisms and their relevant properties, gathering information about the origin, the species where they were found, as well as their mechanism of action and main effects on humans.

## 1. Introduction

Seafood poisoning outbreaks can be originated by marine biotoxins which are naturally produced during harmful algal blooms (HABs). When favorable environmental and climatic conditions coincide, phytoplankton species, mostly dinoflagellates or diatoms, reproduce exponentially and release hazardous toxins. The causes of HABs are still unclear; however, anthropogenic activities and climate changes have contributed to the recent increase in HAB incidence in marine and freshwater ecosystems and at unexpected places [1]. Millions of people around the world need and rely on water resources and services whose availability is strictly dependent on their protection. When toxic episodes start, substantial economic losses occur, and the main sectors affected include public health, commercial fisheries, tourism, recreational activities, monitoring and management. The economic losses caused by HABs in some places and in different sectors have been recently evaluated [2]. In particular, a paralytic shellfish poisoning (PSP) event in New England caused estimated losses of $12 to $20 million in Massachusetts alone, with additional losses in New Hampshire and Maine. Continual PSP intoxication in Alaskan shellfish is one factor blamed for the lack of development of a commercial wild shellfish industry, estimated to be worth $6 million annually [3]. 

Among the thousands of microalgal species known in nature, about 100 produce natural toxins that can cause intoxication or even death in humans and animals [4]. These find their way through the food chain and are subsequently consumed by humans, eliciting diseases or, in the most serious cases, death. Outbreaks of intoxication in humans due to marine biotoxins can have a wide range of symptoms linked to the specific toxic compound. Species belonging to the genera *Alexandrium*, *Gymnodinium*, *Dinophysis*, and *Pseudo-nitzschia* are the main producers of marine biotoxins for humans.

Shellfish, such as mussels, oysters or clams, are filter-feeding molluscs that can accumulate biotoxins according to their natural food chain habits. In all cases, toxic compounds are de novo produced by certain photo- or mixo-trophic microalgae, not by the shellfish, and filter-feeding transfers them to the mollusks, presenting a threat to consumers [5].

These marine animals may store and use a variety of toxins for their own purposes. They vary from small to high molecular weight molecules and display unique chemical and biological features of scientific concern, although most of them are non-proteinaceous compounds. Exposure of consumers to these toxins is a function of the amount of shellfish eaten and the amount of toxin present in the shellfish [6]. As was mentioned, although the majority of these toxins have a phytoplanktonic origin, they are bioaccumulated mainly within the tissues of shellfish after filtering toxic microalgae; this is the reason why they are known as “shellfish toxins”. However, many seafood organisms, apart from bivalve molluscs, can act as toxin vectors, such as echinoderms, tunicates, marine gastropods or crustaceans [7]. To minimize the risk of acute intoxications due to consumption of contaminated species, an appropriate monitoring program must be executed by governments as well as establishing toxins legislation, regulatory limits and reference detection methods.

On the basis of their poisoning symptoms or syndromes, they are classified as toxins causing PSP, amnesic shellfish poisoning (ASP), diarrhetic shellfish poisoning (DSP), neurotoxic shellfish poisoning (NSP), and ciguatera fish poisoning (CFP). However, additional syndromes exist; each type of poisoning is associated with a specific group of biotoxins [8].

According to their own chemical structure, marine biotoxins were classified into eight groups—namely the azaspiracids (AZAs), brevetoxins (BTXs), cyclic imines (CIs), domoic acid (DA), okadaic acid (OA), pectenotoxins (PTXs), saxitoxin (STX), and yessotoxins (YTXs) groups. Two additional groups, palytoxins (PlTXs) and tetrodotoxin (TTX), were also considered.

This article provides an initial survey of phycotoxins usually found in invertebrate animals and their relevant properties, gathering updated research on the origin, the species where they were found, as well as their mechanism of action and main effects on humans. The toxins treated in the article were grouped according to their mechanism of action in the following:

- Neurotoxins acting on the voltage-gated sodium channel, such as BTXs, that cause NSP, saxitoxin (STX), the main compound responsible for PSP, and TTX. BTXs activate site 5 of the α-subunit of voltage-gated sodium channels (VGSCs), while STX and TTX interact with site 1 of these channels. This leads to a blockade of ion conduction and the generation of action potentials, resulting ultimately in loss of neuromuscular function and muscular paralysis.
- Excitatory neurotransmitters, such as DA and analogues, which bind specific receptors in neurons. These toxins are responsible for ASP syndrome, which includes gastrointestinal and/or neurological symptoms [9].
- The rapid-acting CIs, such as gymnodimine (GYMs), spirolides (SPXs) and pinnatoxins (PnTXs), block nicotinic acetylcholine receptors, which again leads to muscular paralysis [10].
- Polyether fatty acid toxins such as OA and dinophysitoxins (DTXs) that have been shown to inhibit protein phosphatases in vitro [11] and are included in the group of DSP toxins.
- PlTXs, large hydrophilic polyalcohols, bind to the plasma membrane Na^+^/K^+^-ATPase, converting the ion pump into a non-specific ion channel, thus allowing the uncontrolled transport of ions across the plasma membrane [12].
- AZAs, PTXs and YTXs, the mechanism of toxicity of which is unknown, are also considered in this paper [6]. This group of toxins was formerly included in the past OA-group toxins; to date, they are separately considered and legislated, and DSP toxins only include OA, DTXs and PTXs in the EU legislation.

In the 21st century, DSP has been the most prevailing poisoning related to marine biotoxins, together with CFP, based on the reported outbreaks that occurred worldwide in the period 2001–2015. More than 1200 recognized cases of intoxication have been reported, most of them in Europe and North/South America, with Chile showing the highest incidence of DSP in Latin America, and also in China; however, it is necessary to mention that the problem is a global issue, and in Africa, parts of Asia and the Middle East, more efforts should be made to implement monitoring programs and risk communication [8].

For the control of phycotoxins, directives and legislations were stated worldwide. Current legal limits established for OA-group toxins by Codex [13], EU [14], Japan [8] and US-FDA [15] are 160 µg OA-eq/kg for DSP toxins and, slightly higher, 200 µg OA-eq/kg in Australia/New Zealand as defined by FSANZ [16]. The same limit applies to AZAs (160 µg AZA1-eq/kg) according to the European legislation, which also sets limits for YTXs, PSP toxins and DA (3.75 mg YTX-eq/kg, 800 µg STX-eq/kg and 20 mg DA/kg) [14].

## 2. Neurotoxins Acting on the Voltage-Gated Sodium Channel

### 2.1. Paralytic Shellfish Poisoning (PSP)

PSP is one of the most studied intoxications with serious symptoms in humans; its causative agents are 58 closely related compounds, whose chemical structure is based on a tetrahydropurine skeleton [17]. In particular, PSP is the result of exposure to STX, a non-terpene alkaloid, and other analogues, such as gonyautoxins (GTXs), neosaxitoxin (NeoSTX), and decarbamoyl-saxitoxin (dc-STX), decarbamoyl-neosaxitoxin (dcneoSTX), decarbamoyl-gonyautoxins (dcGTXs), and the 13-deoxy-decarbamoyl derivatives (doSTX, doGTX), due to the consumption of contaminated shellfish. The constant discovery of new STX analogues is making PSP monitoring a challenging task [17].

#### 2.1.1. Origin

The main producers of PSP toxins are dinoflagellates of the genus *Alexandrium*, *Gymnodinium* and *Pyrodinium*, occurring along the Atlantic and Pacific coast of both Northern and Southern hemispheres [18]; *Gymnodinium catenatum* has also been found in the Mediterranean Sea [19]. To date, PSP toxin-producing species are globally distributed; these genera seem to have expanded during the last decades, so that most coastal countries and, in many cases, large geographic areas are threatened [20].

In addition, some cyanobacteria that may occur in fresh and brackish waters have been reported to produce PSP toxins [21].

#### 2.1.2. Species Where PSP Toxins Were Detected

The major hosts for PSP are the bivalve mollusks, mainly mussels, clams, oysters, scallops and others from many parts of the world [22]. PSP toxins are also found in certain gastropods, crabs and snails which feed on coral reef seaweed and in certain fish. The transvectors accumulate the toxins via feeding in their digestive organs and soft tissues, apparently without harm to them.

#### 2.1.3. Mechanism of Action and Main Effects on Humans

The pharmacological action of PSP toxins is related to the VGSCs, abolishing propagation of the action potential, preventing normal cellular function and leading to paralysis [21].

PSP is characterized by symptoms varying from nausea, vomiting, tingling of the mouth to paralysis, and in severe cases it can be life threatening. This poisoning is due to STX and analogues that bind to VGSCs, inhibiting Na^+^ influx and consequently the generation and propagation of action potentials in excitable cells. This gastrointestinal and neurological syndrome was reported worldwide. The symptoms occurring in the mild form include tingling sensation or numbness around the lips, gradually spreading to the face and neck, a prickly sensation in fingertips and toes, headache, dizziness, and nausea. The moderately severe illness is characterized by incoherent speech, progression of the prickly sensation to arms and legs, stiffness and non-coordination of limbs, general weakness and feeling of lightness, then slight respiratory difficulty and rapid pulse plus backache as late symptoms. In the extremely severe form, muscular paralysis leads to respiratory difficulty, and a choking sensation may occur as well. In fatal cases, death is caused by respiratory paralysis occurring within 2–12 h after the consumption of contaminated shellfish, in the absence of artificial respiration [4]. Many episodes of human intoxication have been reported worldwide at least since the eighteenth century; cases of dead birds, whales or seals were also described [21].

### 2.2. Tetrodotoxin (TTX)

TTX is an extremely potent toxin found mainly in the liver and sex organs of fish, such as puffer fish, globefish, and toadfish and in some amphibian, octopus, and shellfish species. Human poisonings occur when the flesh or organs of the contaminated species are eaten; TTX poisoning can be fatal.

#### 2.2.1. Origin

Pufferfish is the best known source of TTX, although most likely this compound originates from a symbiosis of bacteria (genera *Vibrio*, *Bacillus*, *Aeromonas*, *Alteromonas*, and *Pseudomonas*) with marine animals; moreover, certain phytoplankton species, such as *Alexandrium tamarense* and *Prorocentrum minimum* (*cordatum*), have been reported to be an alternative source [23]. Experimental findings suggested that TTX can be acquired and accumulated from the food chain and that certain species of pufferfish may possess a functional ability to store or eliminate this toxin [24]. It was proposed that the origin of TTX may be due to exogenous, endogenous or symbiotic association among the animals acquiring it and the microorganisms that are reported to produce it [25].

#### 2.2.2. Species Where TTX Was Detected

Most cases of human intoxications by TTX occurred in Japan, through the consumption of pufferfish, which is now forbidden in the European market [8]. TTX was also found in octopus, crabs and gobies from Japan [26]. Nevertheless, TTX and analogues were recently detected in marine bivalves and gastropods from European waters, specifically in a gastropod from Portugal, in mussels and Pacific oysters from England, mussels from Greece and in mussels and oysters from The Netherlands. Therefore, there is strong evidence for the presence of TTXs in European waters [23]. Moreover, the presence of TTX has been recently reported in South and North America due to the consumption of shellfish and fish [8].

The EFSA Panel on Contaminants in the Food Chain studied the levels of TTX found in shellfish from Europe. Results show that TTX concentration in shellfish was low, indicating that there is not a concern for human health due to the consumption of marine bivalves. Nevertheless, the highest concentration of TTX was found in oysters; this could lead to an occasional adverse effect in humans after the consumption of a large portion of oysters (400 g or largest) with high TTX levels [27].

#### 2.2.3. Mechanism of Action and Main Effects on Humans

TTX exerts its toxicity through binding to VGSCs, blocking Na^+^ influx. It induces paralysis of muscles and can be fatal, especially through respiratory failure due to paralysis of respiratory muscles. People intoxicated with these compounds present symptoms within 30 min to 6 h of food ingestion, and after 24 h victims have usually recovered. In the most severe cases, cardiac arrhythmias, muscle paralysis, cranial nerve dysfunction and even death can occur due to respiratory failure [21]. TTX food poisoning is usually reported in Japan, with an average mortality rate of 1.8% from 2008 to 2017: for 332 patients, there were six deaths according to official data by the Japanese Welfare and Labor Administration [8]. In particular, fugu consumption accounts for approximately 50 deaths annually. Apart from intoxication due to consumption of pufferfish, other poisonings occurred due to gastropods and octopus containing TTX [21].

### 2.3. Neurotoxic Shellfish Poisoning (NSP)

NSP is caused by consumption of molluscan shellfish contaminated with BTXs primarily produced by dinoflagellates. Few NSP cases are reported annually; although no fatalities have been described, hospitalizations occur [28].

#### 2.3.1. Origin

BTXs are lipid-soluble cyclic polyether compounds produced by the dinoflagellates *Karenia brevis*, *Karenia brevisulcatum, K. mikimotoi, K. selliformis, and K. papilionacea*, that cause neurologic shellfish poisoning (NSP), particularly in the warm waters of the Gulf of Mexico [28,29]. However, other algae species, such as *Chattonella antiqua*, *Chattonella marina*, *Fibrocapsa japonica* and *Heterosigma akashiwo*, have been reported to produce BTX-like compounds [30]. In addition, *K. mikimotoi* was identified as the likely causative agent, although other suspect species were also present in the bloom which occurred in New Zealand in 1992–1993 [28].

#### 2.3.2. Species Where NSP Toxins Were Detected

BTXs have been reported in many shellfish species, and the most common source of human exposure is through the consumption of clams, oysters and mussels. They were also found in bay scallops, surf clams, southern quahogs, coquinas and tunicates. These compounds were also detected in finfish, although in much lower concentrations [29,31]. Several BTX intoxications were reported in the United States and New Zealand. The largest recorded outbreak of NSP occurred in New Zealand in 1992–1993 due to the consumption of cockles, green shell mussels and oysters [8,21,28]. No intoxication outbreaks in humans or occurrence of BTXs in shellfish or fish have been reported in Europe [32]. Currently, there are no regulatory limits, although the presence of toxin-producing algae, such as *Karenia mikimotoi,* was reported in European waters [33].

#### 2.3.3. Mechanism of Action and Main Effects on Humans

The toxins responsible for NSP are BTXs. They produce an intoxication syndrome nearly identical to that of CFP, in which gastrointestinal and neurological symptoms predominate; NSP is less severe than ciguatera, but nevertheless debilitating, and recovery is generally complete in a few days [29]. Symptoms appear 3–4 h after consumption of contaminated shellfish. Patients complain of non-specific gastrointestinal symptoms (nausea, vomiting, and diarrhea) and neurological symptoms (oral paresthesia, dysarthria, dizziness or ataxia and walking disorders). Patients rarely need hospitalization and supportive treatment, and symptoms typically disappear within 48 h of onset [34], although in extreme cases they may lead to death. The toxins bind to and activate the VGSCs in cell membranes causing depolarization of neuronal and muscle cell membranes [32]. They bind with high affinity to receptor site 5 of the VGSC, leading to activation of these channels with the consequent uncontrolled Na^+^ influx into cells and depolarization of neuronal and muscle cell membranes [21].

In humans, BTXs are also the causative agents of asthma-like symptoms through inhalation exposure [4]; most intoxication occurred through inhalation of aerosolized toxins, especially BTXs, from sea spray exposure.

## 3. Excitatory Neurotransmitters That Bind Specific Receptors in Neurons: Domoic Acid and Analogues

DA is a natural neurotoxin produced by red algae and diatom algal species [35]; it was first isolated in 1958 from *Chondria armata* [36]. Different analogues of DA have been reported, derived from epimerization due to heating, exposure to ultraviolet light and long-term storage. These compounds are heat stable, so cooking does not destroy the toxin [37].

### 3.1. Origin

Various species of red algae and widespread diatoms can produce DA: *Pseudo-nitzschia, C. armata, Digenea simplex* and other related species [35]. Diatoms are distributed in global waters, and they are one of the most morphologically varied and richest phytoplankton groups. Genetic and immunoassay studies led to the discovery of various species of genus *Pseudo-nitzschia* that were related to specific DA outbreaks, but the genetic of DA-producing algal species are currently scarcely known [35]. *Pseudo-nitzschia multiseries* (formally named *Nitzschia pungens f. multiseries*) was suggested as the diatom responsible for the first recognized case of human poisoning in Prince Edward Island, Canada in 1987, during which three people died and more than 150 were affected by the consumption of cultivated blue mussels (*Mytilus edulis*) [35]. In that case, manifestation of neural disorders has been observed in sea mammals and marine birds, as well as humans [36]. DA accumulates in filter-feeding shellfish by consuming DA-producing phytoplankton [35]. Outbreaks occur when populations of DA-producing organisms ‘bloom’ to a sufficiently high concentration to become dangerous to health [36]. The fact that DA-producing algal blooms are accelerating frequently worldwide poses a global health and safety threat and carries exposure risks to a significant number of marine and human lives [35]. The CODEX Committee on Fish and Fishery Products set the maximum limit for DA and its analogues in mollusk flesh for international trade, which is 20 mg/kg [38]. The genus *Pseudo-nitzschia* is a member of pennate diatoms and includes various species described to date. The microalgae form chains of variable lengths with distinct morphological characters, such as needle-shaped and raphid pennate diatoms [35].

### 3.2. Species Where DA Was Detected

The bloom of *Pseudo-nitzschia* and other algal species results in heavy concentrations of DA in global waters, and the toxin is accumulated in shellfish and related animals [35]. The most common bivalves from whom DA has been isolated are mussels (*M. edulis*), razor clams (*Siliqua patula*), clams (*Mya arenaria*), and scallops (*Placopecten magellanicus*). Razor clam and scallop are some of the most significant vectors, as they can hold the toxin for up to one year in the natural environment, or several years after being processed, canned, or frozen [39]. Moreover, DA has also been detected in the Dungeness crab *Cancer magister* and in fish such as anchovies (*Engraulis mordax*) and sardines [9]. These species act as vectors for trophic transfer of DA to a number of marine animals such as sea lions, sea birds, sea otters, whales and also to humans. Other vectors are krill, other mollusks (coastal octopods and cuttlefishes) and planktivorous fish that store significantly high levels of DA in their tissues, but largely in the digestive gland [35].

After the outbreak occurred in Canada, DA is strictly monitored in shellfish sanitary monitoring programs, resulting in a massive reduction of toxic shellfish entering the market. Globally, some shellfish production sites are frequently closed due to the presence of high levels of DA in different species of shellfish. Nevertheless, several scientific reports and papers regularly report that, beside humans, marine wild life is regularly affected by DA intoxication [8]. In early 1993, DA was first detected in shellfish in New Zealand by shellfish monitoring programs [35]. The presence of DA was also documented in Australia, but mainly in North American coasts and in Western Europe [21]. In Europe, prevalence of DA has been reported in wild or cultivated shellfish on the coast of Portugal, Mediterranean regions of France, Italy, Isle of Man (Irish Sea), Galicia (northwest Spain), and Greece [35].

In 2015, the prevalent and most widespread *Pseudo-nitzschia* bloom incident was documented on the Pacific Coast ranging from California to Alaska. High DA levels were recorded in shellfish, resulting in extensive shellfish harvest closures and numerous marine mammal deaths [35]. Within the past 15–20 years, significantly elevated DA levels have been measured on the Pacific coast of the United States; therefore, aggressive monitoring by national and state public health entities appears to have been effective in preventing further deaths by closing shellfish beds if DA levels exceeded 20 mg/kg mollusk flesh [39].

### 3.3. Mechanism of Action and Toxicity on Humans

DA is a water-soluble hapten [35], with three carboxylic acid groups [40], which is chemically derived from an isoprenoid precursor, structurally related to kainic acid [41]. The biological mode of action of DA and its analogues derives from their structural similarity to glutamic acid [36]. They are excitatory amino acids, glutamate agonists [41] which target the kainate receptor (one of three types of ion channels) present in various vital organs [35] and cause neuronal depolarization [36]. The potency of neuroexcitation depends on the strength of binding [36]. The biological effects of kainoids have long been exploited therapeutically for their insecticidal and anthelmintic properties [36].

A cascade of neural disorders characterized the poisoning: memory impairment, recurrent seizures, and epilepsy [35]; short-term memory loss is a typical symptom, which led to the name ASP [36]. Dizziness, nausea and vomiting are other symptoms, ultimately leading to coma and brain damage or death in the most severe cases [36]. People affected by the poisoning which occurred in Canada showed gastrointestinal, cardiovascular, and neurologic disorders and permanent short-term memory loss. In fact, DA affects the central, peripheral, and autonomous nervous system, and skeletal and smooth muscle [35]. Thus, DA neurotoxicity potentially may be associated with a non-amnesic syndrome [39].

Although having a short life span in various tissues, DA causes severe pathological alterations in vital body organs; it penetrates the blood–brain barrier, threatening neurons and glia. DA induces intracellular free radical generation at the level of the mitochondria, and its accumulation leads to the oxidation of vital macromolecules including lipids, proteins and DNA. This phenomenon is referred to as oxidative stress and can induce apoptosis or necrosis of neurons and glia. Lesions in the human brain, particularly in the hippocampus, have been reported in human ASP cases. Neuronal necrosis compromises the physiology of the central nervous system, including motor, sensory and cognitive deficits, and causes psychological alterations. The behavioral changes are similar to the diagnostic features of schizophrenia and anomalies in social behavior that are related to autism spectrum disorder [35]. DA poisoning results in three progressive stages: the first is characterized by the specific appearance of epileptic lesions; then, alterations of physiology and physical damages of the organs; and finally, progressive damage with recurrent seizures occurs [35]. By penetrating the protective placental membranes, DA causes detrimental physiological and structural effects on the fetus, with highly persistent alterations on brain development [35].

Chronic low level exposure by DA is thought to impact human health; milder memory problems may be associated with the large consumption of contaminated seafood, especially of razor clams [39].

## 4. Toxins Acting on Nicotinic Receptors: Cyclic Imines

CIs are macrocyclic compounds, produced by dinoflagellates, which share an imine functional group within their chemical structure [42]. SPXs, GYMs, PnTXs, pteriatoxins (PtTXs), prorocentrolide, portimine and symbioimine belong to this group of lipophilic toxins.

### 4.1. Origin

SPXs, produced by the dinoflagellates *Alexandrium ostenfeldii* and *Alexandrium peruvianum* [43,44], were discovered in the 1990s from mussels (*M. edulis*) and scallops (*P. magellanicus*) during a routine monitoring of the lipophilic DSP toxins in the Atlantic coast of Nova Scotia, Canada [45]. Nowadays, 16 SPX analogues have been isolated from shellfish and phytoplankton extracts in European, North American, and South American coasts [46,47].

GYMs were first isolated from oysters (*Tiostrea chilensis*) from the South Island of New Zealand [48,49]. This original isolation was linked to a concurrent bloom of the gymnodinoid dinoflagelate *Gymnodinium* cf. *mikimotoi*. Later, the dinoflagellate *Karenia selliformis* was confirmed to produce the analogues GYMs B and C [50,51]. On the other hand, the dinoflagellate *Alexandrium peruvianum*, responsible for the 13-desmethyl spirolide production, was reported to produce a novel gymnodimine analogue, 12-methylgymnodimine [52], suggesting that common biosynthetic pathways exist between *Karenia selliformis* and *Alexandrium* *peruvianum* dinoflagellates.

The organism responsible for PnTXs, the dinoflagellate *Vulcanodinium rugosum*, was discovered after the analysis of sediment samples from Rangaunu Harbour and the French Mediterranean coast. The species was also found in South Australia, China, Spain, Hawaii and Japan [53,54,55,56,57,58]. The first analogue of this group to be discovered was PnTX A and was isolated from the digestive gland extract of *Pinna attenuata* in China and Japan. PnTXs B, C and D were isolated from viscera of the *P. muricata* [59,60,61], meanwhile PnTXs E and F were found in the Pacific oysters (*Crassostrea gigas*) from Ranganau Harbour, Northland, New Zealand [62]. On the other hand, PnTXs E, F and G have also been isolated from Pacific oysters and razorfish (*P. bicolor*) from South Australia [63,64] and from the Norwegian blue mussel (*M. edulis*) [65]. PnTXs A, B and C were isolated in 2001 by Uemura and co-workers from *Pteria penguin* [61]. There is still no conclusive evidence for the natural source of these compounds, and it is not clear whether they are produced by algae or by metabolic modification of other PnTXs. PtTXs are thought to be produced by bio-transformation reactions of a precursor PnTX G too [64].

The prorocentrolide and the spiro-prorocentrimine were detected in related dinoflagellates, as *Prorocentrum sp.*, a neritic species that can be found in both temperate as well as tropical oceans. In 1988, prorocentrolide was detected in *Prorocentrum lima* [66]. In addition to these subgroups of CIs, other two natural products include portimine, and symbioimine that have been isolated from the dinoflagellates *Vulcanodinium rugosum* [67] and *Symbiodinium* sp. [68].

### 4.2. Species Where CIs Were Found

The dinoflagellates *Karenia selliformis*, *Alexandrium ostenfeldii*, *Alexandrium peruvianum* or *Vulcanodinium rugosum* are nutrients for filter-feeding bivalve mollusks, crustaceans, or finfish. These species may proliferate under favorable environmental conditions, producing CIs. In fact, there is a global increase of these HABs worldwide and the causes remain unexplained [69]. However, eutrophication, ballast water introduction or climate change have been associated to these increasing new algal phenomena [1].

SPXs had been reported in different mollusks such as mussels (*M. edulis*, *M. galloprovincialis*), scallops (*P. magellanicus*), razor clams (*Ensis arcuatus*), oysters (*C. gigas*), clams (*Mulinia edulis*) or in macha (*Mesodesma donacium*) harvested from different regions of Europe (Norway, Spain, Italy, France...), in Canada, New Zealand, Chile or China [45,70,71,72,73,74,75,76,77,78,79]. 

GYMs had been found in green shell mussel (*Perna canaliculus*), mussels (*M. galloprovincialis*), dredge oysters (*T. chilensis*), scallops (*Pecten novaezelandiae*), pipi surf clam (*Paphies australis*), paua or abalone (*Haliotis iris*) in New Zealand, clams (*Ruditapes decussatus*) harvested in Tunisia [80], pipis (*Donax deltoides*), mussels (*Modiolus proclivis*) and in oysters (*Saccostrea glomerata*) in Australia [81], and *C. gigas* from South Africa [82] or in Chinese shellfish [79].

PnTXs have now expanded to Pacific oysters (*C. gigas*), mussels (*M. galloprovincialis*), razor fish (*P. bicolor*) or clams (*Venerupis decussata*) from Japan, China, South Australia, New Zealand, Canada, Norway, France or Spain [58,64,65,83,84,85,86].

PtTXs were first reported in the bivalve *P. penguin* [61], together with Prorocentrolides and spiro-prorocentrimine, isolated from phytoplankton species. They have not been reported in any other species until day.

### 4.3. Mechanism of Action and Main Effects on Humans

CIs are known as “fast-acting” toxins because they induce rapid death in intraperitoneal (i.p.) mouse bioassay [87]. Despite some reviews about their toxicity which have been undertaken [85], no further information is available regarding chronic toxicity data and no adverse effects are reported in humans following consumption of shellfish containing CIs [88].

The 13-desmethyl SPX-C, SPX-C and 20-methyl SPX-G are the most toxic SPXs after i.p. injection, with LD50 values of 6.9–8.0 µg/kg body weight (b.w.) [88]. The neurotoxic symptoms described in mice include hunched appearance, abdominal breathing, respiratory distress, contractions, tremors and death within 3–20 min after receiving lethal doses of SPXs [89]. On the other hand, GYM A was highly toxic to mice following i.p. injection with LD50 ranging values of 80–96 µg/kg b.w. In this case, the neurotoxic symptoms described include hyperactivity, jumping, paralysis, extension of the hind legs and even death after 15 min of injection [88]. The analogue GYM A is reported to be 10 times more toxic than GYM B [90]. For a mix of PnTX E and F, the LD50 values were 23 µg/kg b.w. and 60 µg/kg b.w., respectively, following administration in food, recording the lowest values of any of the CIs [87]; meanwhile, the limited data available for a PtTX B/C mix showed that this was 12 times more toxic than PtTX A [61].The isolation and purification of the macrolide spiro-prorocentrimine from a culture of *Prorocentrum* species from Taiwan shows an LD99 value of 2.5 mg/kg in mice after the i.p. injection [91]. No toxicity data has been published for prorocentrolide [88]; nevertheless, following the structural elucidation of the spiro-prorocentrimine using X-ray crystallography and nuclear magnetic resonance (NMR), similar structural features were reported in comparison with prorocentrolide [92]. The i.p. mouse toxicity LD50 was 1570 μg/kg in the case of the portimine, indicating a much lower toxicity than many other cyclic imine shellfish toxins. However, this compound was highly toxic to mammalian cells in vitro with an LC50 to P388 cells of 2.7 nM, with activation of caspases indicating apoptotic activity [47].

The mechanism of neurotoxicity of both SPXs and GYMs is based on the inhibition of muscarinic (mAChRs) and nicotinic acetylcholine receptors (nAChRs) [88,93], with a reversible effect in the case of GYM [42,94]. Moreover, a recent work specifies that the toxicity of SPXs is due to their high inhibition potency on different peripheral and central nAChRs subtypes [95]. There are currently no data on the mode of action of other CIs, but it is thought that PnTXs also target nAChR [64,96].

On the other hand, there is no information on the absorption, distribution or excretion of SPXs, GYMs or PnTXs in animals or humans [87], but information based on oral administration [88,97,98] indicates that these compounds are absorbed from the intestinal tract from where they reach different organs [99].

## 5. Polyether Toxins: Okadaic Acid, Azaspiracids, Pectenotoxins

### 5.1. Okadaic Acid Group

OA is a polyether containing a carboxylic acid group and three spiro-keto ring assemblies, one of which connects a ring of five members with a ring of six members. There are different types of esters of OA and DTXs: these are fatty acid esters of OA, dinophysitoxin-1 (DTX1) and dinophysitoxin-2 (DTX2), with variable chain lengths and collectively known as dinophysitoxin-3 (DTX3). These compounds are potentially present in shellfish as a result of toxins metabolic pathways.

#### 5.1.1. Origin

OA was initially isolated from marine sponges *Halichondria okadai* from the Pacific coast of Japan, and *Halichondria melanodocia*, in Florida Keys [100]; afterwards, it was isolated from the dinoflagellate *Prorocentrum lima* [101]. The first toxin of the OA group to be characterized was DTX1, and it was linked to DSP [102]. It was isolated in Japan from the hepatopancreas of mussel *M. edulis*, and its production was attributed to *Dinophysis fortii*. The third main analogue, DTX2, was discovered in Irish mussels associated with DSP [103]. Then, OA and DTXs are produced by *Dinophysis* and *Prorocentrum* species.

#### 5.1.2. Species Where OA and DTXs Were Found

These lipophilic marine toxins are accumulated by shellfish, mainly bivalve. Mussels have been responsible for human intoxications consisting of gastrointestinal disorders, reported in the Netherlands since 1961 and later on in 1971, 1976, 1979 and 1981 after consumption of *M. edulis*, previously exposed to a phytoplankton bloom of the dinoflagellate *Dinophysis acuminata* [104]; other poisoning events occurred in Los Lagos, Chile in 1970 [105], and in Japan in 1976 and 1977 after the ingestion of mussels (*M. edulis*) and scallops (*Patinopecten yessoensis*) [102]. Other outbreaks after mussels (*M. galloprovincialis*) consumption were reported in Galicia, Spain, in 1978, 1979, and an especially relevant one in September 1981 [106]; mussels *M. edulis* consumption was also the cause of intoxication in France in 1983 [107], Sweden and Norway in 1984 [108,109]. There were also toxic outbreaks due to clams (*Donax trunculus*), and razor clams (*Solen marginatus*) consumption in London and Portugal between 1997 and 2001 [110,111]. More outbreaks due to mussels occurred in Greece in 2006–2007 [112], and in Canada and China in 2011 [113,114,115].

Crustaceans have been shown to accumulate DSP toxins in Europe, namely esterified derivatives of the OA DTX3 [7]; it was found in green crab (*Carcinus maenas*) in Portugal [110]. It was also described an intoxication in Norway after consumption of brown crabs (*Cancer pagurus*), possibly accumulating toxin metabolites from blue mussels ingestion [116].

The trophic transfer of lipophilic toxins is considered to be limited to upper trophic level. These toxins are biotransformed mainly in filter feeder organisms, producing ester metabolites of the OA and spirolide toxins groups, which have also been found in different trophic levels in the North Sea [117]. An evaluation of the levels of multi-toxin mixture in the marine organisms might be crucial to assess their bio-transference through the trophic chain, as well as risks in human health. In this context, fatty acid acyl esters of OA and DTX2 were found in the stomach and liver of mackerel from the North Sea [117].

#### 5.1.3. Mechanisms of Action and Main Effects on Humans

The target of OA and analogues was suggested to be the serine/threonine phosphoprotein phosphatases (PPs), in particular PP2A, and as secondary targets, PP1 and PP2B [118,119]. They induce the disruption of duodenal paracellular permeability due to altered tight junction integrity [120]. The symptoms caused by OA group toxins are diarrhea, nausea, vomiting and abdominal pain, and thesemay occur in humans shortly after consumption of contaminated bivalve mollusks. Inhibition of PPs is assumed to conform their mode of action [11]. However, recent investigations have shown that both the target and the mode of action of this group could differ from what has been previously assumed [121,122,123]. The precise toxicological mechanism of OA resulting in diarrhea and its subsequent effects in mammals has not been established. Further study of the toxic mechanisms, particularly at the proteomic and genomic levels, could help to elucidate the precise toxicological mechanism of OA in vivo [124]. Proteomic analysis has shown that OA toxicity destroyed the digestive enzyme system, affecting lipid, amino acid and sugar metabolism, cytoskeleton reorganization, and inducing oxidative stress, interfering with the cell signal transduction in intestinal cells. These observations evidenced that OA toxicity in intestines is complex and diverse, and multiple proteins and biological processes are involved in the diarrheic process [122].

Regarding the mechanism of action of OA, the potent inhibition on PPs has been linked to its acute toxic effects, its tumor promoting activity and its neuronal toxicity, in addition to the described intestinal disorders. However, some studies suggest that the evidence in support of such association is very limited [123]. The pathway linking the enzyme inhibition to toxic effect has not been identified, and there is no proportionality between the severity of the toxic effects of the OA and its inhibitory activity. Furthermore, it has been remarked that substances that are not PPs inhibitors can induce the same toxic effects as OA and derivatives, and these toxic effects in animals cannot be reproduced consistently by other well-known PPs inhibitors [123].

### 5.2. Azaspiracids

AZAs were first detected as causative compounds in a new type of food poisoning after ingestion of mussels harvested from Ireland and consumed in the Netherlands in 1995 [125]. AZAs were responsible for the azaspiracid poisoning (AZP) syndrome. The AZAs were originally classified together with DSP toxins, owing to the similarities in gastrointestinal symptoms [126]. However, no indication of PPs inhibition was demonstrated [127,128], and at present, AZAs are classified as a separate group of toxins.

The structure of AZAs consists of a cyclic amine, the tri-spiro-assembly, and the carboxylic acid group. Several compounds have been isolated from mussels: mostly AZA1, AZA2 and AZA3, differing in the number of methyl groups. This group is composed of at least 30 analogues [126].

#### 5.2.1. Origin

The genera of AZA-producing phytoplankton are *Azadinium* and *Amphidoma* [129]. AZA1 is the main compound found in mussels from Ireland, isolated in 1998 [130], although its structure was fully elucidated later by synthetic studies [131]. Seafood contamination with AZAs has been reported in different locations from Europe, North and South America, Africa, and Japan [21,132], and new AZAs have been recently described [133,134].

#### 5.2.2. Species

Concerning AZAs, blue mussels (*M. edulis*) from Ireland were the main organisms containing these compounds and resulting in AZP in humans [135,136]. Other shellfish species, such as oysters (*C. gigas* and *Ostrea edulis*), Chilean mussels (*Mytilus chilensis*), razor fish (*Ensis siliqua*) and scallops (*Pecten maximus* and *Argopecten purpuratus*) were also reported to contain AZAs [137,138,139]. AZAs occurred in crustaceans, such as crabs (*C. pagurus*), feeding on contaminated mussels, however, no intoxications in humans after crabs consumption have been reported [140].

#### 5.2.3. Mechanisms of Action and Main Effects on Humans

AZAs act on several known targets; however, their mechanism of action is not yet known [141]. AZAs were shown to be cytotoxic, affect cytoskeleton arrangements, and inhibit potassium channels (hERG—human ether-à-go-go-related gene), important in the cardiac action potential, causing cardiac toxicity [142]. Damage to multiple organs was produced after oral administration to rodents, affecting the intestinal epithelium, *lamina propria* and villi [143]. In humans, they cause vomiting, nausea, diarrhea and stomach cramps, starting a few hours after ingestion and lasting up to 30 h, with full recovery after 2–3 days [136]. EFSA established an acute reference dose (ARfD) of 0.2 μg AZA equivalents/kg b.w. [144]. Neurotoxicity linked to AZAs [145] could be explained by the fact that a combination of AZAs and glutaric acid inhibits VGSCs, together with the surprising observation that AZAs occur only in mussels with high levels of glutaric acid [146].

AZP has emerged recently in France and Belgium [8,135] and in Washington, USA [136] after consumption of blue mussels from Ireland.

### 5.3. Pectenotoxins

PTXs take their name from the organism where they first were discovered: the digestive gland of Japanese scallop, *P. yessoensis* [147]. These toxins are heat-stable polyether macrolide compounds, with structures containing a spiroketal group, three oxolanes, a bicyclic ketal and a six-membered cyclic hemiketal [148]. PTX2 is believed to be the main precursor originating other analogues after biotransformation during metabolic processes in bivalves.

PTXs usually co-occur with the OA-group of toxins, and they are currently included in the regulatory limit for the mentioned group. However, they do not exhibit the same mechanism of action, and EFSA recommended not including them in the regulatory limit for the group of OA toxins [11]. At present, in the European Union, they are considered in the same group for regulatory purposes (EU Regulation 853/2004) [14]. 

#### 5.3.1. Origin

The dinoflagellate *Dinophysis fortii* was at first identified as the producing organism [149], while it was discovered later by different researchers that PTXs were also present in other dinoflagellates: *D. acuminata*, *D. acuta*, *D. caudata*, *D. rotundata* and *D. norvegica* [150]. PTX1 and PTX6 are the main compounds found in the Japanese scallop, and PTX2 seco acid (PTX2 SA) and its epimer 7-epiPTX2 seco acid (7-epi-PTX2 SA) are found in mussels and scallops. Other isomers of PTX1 and PTX6 have been identified: PTX4 and PTX7 [151], and later on, PTX11 [152]. Fatty acid esters of the analogues PTX2 SA and 7-epi-PTX2 SA are formed during metabolism in shellfish.

#### 5.3.2. Species Where PTXs Were Found

PTXs were found in Japanese scallops (*P. yessoensis*), and in different bivalve mollusks from diverse geographic origins: mussel species *P. canaliculus* and *M. edulis*, scallops *P. novaezelandiae* and *P. yessoensis* and cockles *Cerastoderma edule* from Japan, different European countries and New Zealand among others [153,154,155,156,157].

#### 5.3.3. Mechanisms of Action and Main Effects on Humans

PTX1 and PTX2 have been described to alter F-actin in several cell types [158,159,160,161]. The structure-activity studies show that the molecule ring plays a key role in actin binding [148]. No human illness associated to exposure to PTXs has been reported.

## 6. Toxins That Act on Ion Pumps or Channels: Palytoxin (PlTX) and Derivatives

PlTX was first isolated in the early 1970s in Hawaii from the soft coral *Palythoa toxica* [162], but it was subsequently detected in many other species belonging to the genus *Palythoa*, such as *P. aff. margaritae* [163], *P. vestitus* from Hawaii [164], and *P. mammillosa* and *P. caribaeorum*, both collected from the coral reefs of the Caribbean Sea [165,166,167,168]. This compound is a large and very complex molecule with both lipophilic and hydrophilic regions and has the longest chain of continuous carbon atoms in any known natural product [169].

### 6.1. Origin

PlTXs are known to be produced by the epi-benthic or epi-phytic dinoflagellates of the genus *Ostreopsis*, which has a wide global distribution in temperate and tropical waters [170]. The compound isolated from a Japanese strain of *O. siamensis* was named ostreocin-D. Other species from the genus *Ostreopsis* were later found to contain other PlTX-like compounds, such as *O. mascarenensis*, containing mascarenotoxins [171], or *O. ovata*, a source of ovatoxins [172].

In order to explain PlTX presence in different species, some authors proposed bacteria as producing organisms. PlTX-like compounds were detected in Gram-negative *Aeromonas* sp. and *Vibrio* sp. bacteria using anti-PlTX antibodies [173] and PlTX and 42-hydroxy-PlTX were isolated from marine *Trichodesmium* spp. cyanobacteria [174].

Other structurally-related compounds have been identified from other *Palythoa* sp. extracts, and the number of known PlTX-like analogues now approaches 20, including the structurally-related ostreocin-D, ovatoxins a–k and isobaric palytoxin [170,172,175,176,177,178,179].

### 6.2. Species Where PlTXs Were Found

The vectors of PlTXs are mainly crabs (*Demania reynaudii*) [180], parrotfish (*Scarus ovifrons*) [181], goldspot herring (*Herklotsichthys quadrimaculatus*) [182,183,184], and serranid fish (*Epinephelus sp*.) [185]. Moreover, the popularity of home aquaria containing living corals has increased, causing concern for the important impact on human health associated with the manipulation and maintenance of these corals. There are different species of decorative soft corals, such as *Sarcophyton*, *Sinularia*, *Nephthya*, *Cladiella*, *Xenia*, Palythoa, and *Zoanthus* species [186]. The species belonging to the genera *Palythoa* and *Zoanthus* are widely used due to their colorful and ornamental features [187,188] and these are known to accumulate PlTX or its analogues such as 42S-OH-50S-PlTX isolated from *P. toxica* [189], 42S-OH-50R-PlTX identified in *P. tuberculosa* [190], and deoxy-PLTX isolated from *P. heliodiscus* [187].

### 6.3. Mechanism of Action and Main Effects on Humans

Many efforts have been devoted to defining the mechanism of action of PlTXs. Pharmacological and electrophysiological studies have demonstrated that PlTXs act as a haemolysin altering the function of excitable cells. These compounds selectively bind to the Na^+^, K^+^-ATPase [191] and transform the pump into a channel permeable to monovalent cations [192,193,194,195]. The consequent increase of intracellular Ca^2+^ concentration ([Ca^2+^]) stimulates the release of neurotransmitters by nerve terminals, of histamine by mast cells, and vasoactive factors by vascular endothelial cells as a signal. It also induces contractions of striated and smooth muscle cells. Some other effects due to a rise in [Ca^2+^] may be the activation of phospholipase C and A2 [192,196]. Moreover, there are various reports that propose PlTXs opening an H^+^ conductive pathway resulting in activation of the Na^+^/H^+^ exchanger [197,198]. Other authors suggest that PlTXs raises [Ca^2+^] independently of the activity of voltage dependent Ca^2+^ channels and Na^+^/Ca^2+^ exchange [199]. Damages at the cytoskeleton level induced by PlTXs, such as depolymerization of actin filaments in intestinal and neuroblastoma cells, were also reported [200,201,202].

Cases of human poisonings ascribed to PlTXs have been associated with oral, cutaneous, inhalational and ocular exposure routes, with oral exposure after the ingestion of contaminated fish or crustaceans the most harmful for human health. A limited number of foodborne poisonings have been documented in tropical and subtropical regions [180,181,182,184,185]. Some of the main symptoms of poisoning are nausea, diarrhea, and vomiting with convulsions, dizziness, numbness, and restlessness in some cases, and subsequently weakness, muscle cramps, myalgia, rhabdomyolysis, bradycardia, tachycardia, respiratory failure and even death in the worst cases [184,203].

On the other hand, in temperate areas, human incidents were associated with inhalation of marine aerosol and/or cutaneous exposures to seawater during *Ostreopsis* blooms. In these cases the most common signs were respiratory distress, rhinorrhea, cough, fever, and dermatitis [204,205,206,207,208,209].

In the last years, the evidence points to the idea that inhalational and/or cutaneous exposure to PlTXs could also occur after handling contaminated soft corals during maintenance of home marine aquaria. The toxic potential of PlTXs identified in soft corals raise a serious concern for human health, due to the growing number of documented cases.

## 7. Unknown Receptors: Yessotoxins

YTXs are disulfated polyciclic ether compounds structurally related to BTXs and CTX [210,211]; they were included in the list of marine toxins due to the coexistence with diarrheic toxins and the lethality on mice after i.p. injection [212].

### 7.1. Origin

This group of naturally occurring toxins is produced by the dinoflagellates *Protoceratium reticulatum* [213], *Lingulodinium polyedrum* [214] and *Gonyaulax spinifera* [215]; the bloom of these algal species make their toxins accumulate in edible tissues of filter feeding shellfish and then enter into the food chain [211].

The exact number of YTXs and the chemical structure of most of them have not been determined yet, even though the presence of almost 100 analogues has been reported from bivalves and dinoflagellates [211]. As generally occurs for natural contaminants, some YTXs are directly produced by dinoflagellates, while others are produced by the shellfish metabolism. More than 90 YTXs found in dinoflagellates derive from *P. reticulatum* [210]. The ecological role of these compounds is already unknown [212].

### 7.2. Species where YTXs Were Found

The lead compound of the group, yessotoxin (YTX), was first isolated in 1986 from the digestive glands of scallop *P. yessoensis* collected in Mutsu Bay (Japan), from which the toxin acquired its name [216]. Thereafter, YTXs have been detected worldwide, in shellfish from Korea [217], Chile, and New Zealand. In Europe, they have been described in mollusks from Norway, Italy, Spain, and Russia [212].

### 7.3. Mechanism of Action and Toxicity on Humans

The precise mechanism of action of YTXs is still unknown [210]; these toxins were generally detected together with other lipophilic toxins of the DSP group, such as OA, during the shellfish extraction procedure. For this reason, YTXs were initially included in this toxins group [211]. However, YTXs do not share the same biological activity (diarrheogenic effects), since the toxic activity (inhibition of PPs) is four orders of magnitude lower [21]. Therefore, the European Commission classified and regulated them separately from the diarrheic toxins [218].

Although the contamination of shellfish from YTXs is reported worldwide, sometimes at concentrations up to mg/kg, no human poisonings were described in literature, and symptoms of intoxication produced by YTX in humans are relatively unknown [8,210,211].

From in-vivo experimental toxicity studies, the target organ of YTX and some analogues, homoyessotoxin and 45-hydroxy-homoyessotoxin, seems to be the heart; in particular, cardiac muscle cells. The pathogenetic mechanism of the cardiac toxicity is still not completely understood, though in-vitro studies ascertained the cytotoxic activity of YTX through the alteration of intracellular levels of calcium and cyclic AMP, cytoskeletal modifications, caspases activation and the opening of the permeability transition-pore of mitochondria [211]. The chemical structure of YTX, being similar to that of BTXs, suggests a possible action of the toxin against VGSC activity [210]. However, some studies demonstrated that the effect of YTX on cytosolic calcium levels is a direct consequence of calcium channel activation and is not linked to sodium channels [219]. In fact, some investigations focused on the modulation of Ca^2+^ homeostasis in human lymphocytes by YTXs through the activation of phosphodiesterases [220,221,222]. This mechanism of action would explain the cardiotoxicity of these toxins [210]. Moreover, YTXs showed activity on the protein kinase C translocation in primary cortical neurons [223]. Therefore, YTXs should be considered as potentially toxic for humans, since they were reported to produce neuronal damage at brain level [210]. On the other hand, it has been demonstrated that the analogue di-desulfo-yessotoxin induces fatty degeneration in liver and pancreas [211]. At any rate, short term and chronic toxicity data are not available and pharmacokinetic studies are lacking [211].

Since no cases of human intoxication have been reported, the European Union recently increased the limit of YTXs in shellfish from 1 to 3.75 mg of YTX equivalent/kg of shellfish meat as a preventive measure [218]. However, according to some authors, for a comprehensive and correct risk evaluation of YTXs, the intake of contaminated seafood by the sensitive population (such as heart patients) should be taken into account. Since OA has been often detected in YTX-contaminated shellfish, the effects of the concurrent presence of YTXs and OA, as well as of other naturally occurring cardiotoxic agents, have to be carefully investigated [211].
